# 

**Published:** 1898-03

**Authors:** 


					CONTENTS.
PAGE)
In Memoriam:
Augustin Prichard,
F.R.C.S. Eng., M.D. Berlin, L.S.A.
The Treatment of Enteric Fever.
By
J. Michell Clarke, M.A., M.D.
Cantab., F.R.C.P
IS
On Some Diseases Occurring in Men
Who Worked in a Sewer.
By
F. H. Edgeworth, M.B., B.A.
Cantab., B.Sc. Lond
33
Progress of the Medical Sciences:
Medicine.
Theodore Fisher, M.D.
33
Surgery.
W. M. Barclay
45
Progress of the Medical Sciences
(continued):
Ophthalmology.
Cyril H. Walker,
M.B.
50
Reviews of Books
ss
Notes on Preparations for the Sick
Meetings of Societies
84
The Library of the Bristol Medico-
Chirurgical Society  90
Local Medical Notes
94
Scraps
95

				

